# National Surveys of Population Health: Big Data Analytics for Mobile Health Monitors

**DOI:** 10.1089/big.2015.0021

**Published:** 2015-12-01

**Authors:** Bruce R. Schatz

**Affiliations:** Department of Medical Information Science, Institute for Genomic Biology, University of Illinois at Urbana-Champaign, Urbana, Illinois.

**Keywords:** big data analytics, big data architecture, crowdsourcing, population health, predictive analytics

## Abstract

At the core of the healthcare crisis is fundamental lack of actionable data. Such data could stratify individuals within populations to predict which persons have which outcomes. If baselines existed for all variations of all conditions, then managing health could be improved by matching the measuring of individuals to their cohort in the population. The scale required for complete baselines involves effective National Surveys of Population Health (NSPH). Traditionally, these have been focused upon acute medicine, measuring people to contain the spread of epidemics. In recent decades, the focus has moved to chronic conditions as well, which require smaller measures over longer times. NSPH have long utilized quality of life questionnaires. Mobile Health Monitors, where computing technologies eliminate manual administration, provide richer data sets for health measurement. Older technologies of telephone interviews will be replaced by newer technologies of smartphone sensors to provide deeper individual measures at more frequent timings across larger-sized populations. Such continuous data can provide personal health records, supporting treatment guidelines specialized for population cohorts. Evidence-based medicine will become feasible by leveraging hundreds of millions of persons carrying mobile devices interacting with Internet-scale services for Big Data Analytics.

## Introduction

Healthcare is the economic crisis of our time, yet there is no viable infrastructure for health systems due to the shift from acute care to chronic care.^1^ At the core is a fundamental lack of actionable data; it is not possible today to stratify individuals to predict which persons have which outcomes within whole populations. Increased quality at decreased cost can be achieved by accurately placing persons into population cohorts with each cohort treated optimally.

A new health system with better health management will require better health measurement.^2^ New technologies can now provide the rich data sets necessary for adequate health measurement, which can enable predictive modeling for improving practical healthcare. These technologies leverage the mass commercialization of mobile devices and Internet services, such as smartphones and cloud clusters. However, their utility is unproven in health applications, particularly at the scale needed for population health measuring human variation of physiological and psychological parameters.

National Surveys of Population Health (NSPH) involve measuring large numbers of people. Traditionally, they have been focused upon acute medicine, measuring people to contain the spread of epidemics. After World War II, epidemiology began to shift to include chronic conditions as well. The turning point was the development of the risk factor in the Framingham Heart Study (FHS).^3^

Framingham is a small town in Massachusetts outside of Boston. In the early 20th century, it was the site of a major trial for tuberculosis control sponsored by MetLife. The US Public Health Service in 1948 began a 20-year study into risk factors for heart disease. The study enrolled a group of town persons into a program to be measured annually using medical tests from examination to laboratory to questionnaire.^4^ The FHS discovered strong correlations between lifestyle behaviors and heart disease, most notably the relationship with blood pressure, within this longitudinal epidemiological study.^5^

FHS pioneered the paradigm of risk factor, of health features correlated with chronic disease. However, correlations could only be made between heart disease and the features that were being measured. For example, blood pressure was measured during examinations, so correlations could be made. However, social stress was not measured due to difficulty of measurement, although it was known that this might be a stronger determinant of heart health than blood pressure.^5^

**FIG. 1.**
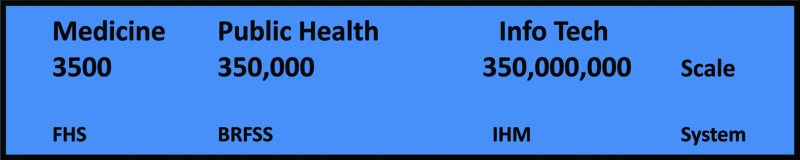
Scale of persons for population health as measured by different technologies. Medicine uses human examination (e.g., Framingham Heart Study), Public Health uses telephone calls (Behavioral Risk Factor Surveillance Survey), and Info Tech uses network computing (Internet Health Monitors).

Due to logistics of manual examinations, the demographics of Framingham were limited. The population studied was homogeneous middle-aged whites in a small New England town. A full study would need to encompass demographic variation, of age and sex and race and region. Daily life would be measured, including conditions well beyond heart disease. The scale of seeing patients with medical examinations by physicians was about 3500 persons longitudinally, as compared in Figure 1.

The primary need in measuring data for population health is reference data sets for large diverse populations. Such data sets would revolutionize healthcare with big data by providing more accurate measures of which features are correlated with which outcomes. Hundreds of years of acute care have produced baselines of safe ranges of measured values. However, chronic care is recent enough for large populations that such baselines do not exist yet at present. The rise of personalized medicine (sequenced genomes) has not produced common gene networks discriminating major chronic conditions, nor has the rise of quantified self (fitness devices) produced common heart rate variations predicting major potential risks.

To support population stratification, longitudinal studies are essential for establishing standard baselines. It is not necessary to perform prospective epidemiology, where the clinical trials must wait for participant incidence of a particular disease. An effective combination of mobile devices and Internet services can continuously monitor whole populations across full lifestyle features. A national survey of measuring data for population health would provide a unique resource for many analyses, pioneering correlations of everyday health, just as the Framingham Study pioneered correlations of heart disease with a smaller longitudinal sample. Moving beyond the current scale of demographic sampling would effectively determine the health of populations.^6^

## Present Technologies Using Quality of Life Questionnaires

The major current method for population health measurement is questionnaires, self-administered assessment questions measuring quality of life (QOL). The idea is that the patient is asked a series of questions, centered on certain themes, which can be reliably answered to assess health status. The questions are asked in a short session, typically 10–15 minutes, so the number of questions is few, typically 20–30. Including disease-specific questionnaires, there are literally a thousand QOL instruments.^7^

There are nearly 100 general health status questionnaires, where the patients fill out a short paper form to record their health status. The best-known general health questionnaire is the SF-36 (Short Form, 36 questions), which grew out of work at the Rand Corporation in the late 1970s and 1980s. The Rand researchers, inspired by the concept of a health index, searched for a means to determine patient outcomes from disease and treatment, as well as a means to monitor a specific disease. Their summary book, *Measuring Functioning and Well-Being: The Medical Outcomes Study Approach*, indicates their paradigm.^8^

The original Medical Outcomes Study in the 1980s went systematically through all the determinants of health.^9^ This study produced a master list of hundreds of questions that covered all aspects of QOL. Note that because of their orientation for medical outcomes after clinic visits, the questions focused primarily on physical and mental measures rather than social and societal measures. If computer technology had then been widely available, they would have directly used this master list with all the questions. Due to the technological limitations at that time, they instead chose the medium of paper forms, which forced them to statistically choose the most discriminating questions.

Health status questionnaires have been shown to be effective for predicting patient outcomes to treatment for severe conditions. For example, there was a large VA clinical trial of 2885 patients using the SF-36 as a screening tool for patients about to undergo heart surgery. The question scores were correlated with survival outcome, with a strong relationship between self-rated health and subsequent coronary artery disease-related mortality. The elderly patients' self-ratings of health were more accurate in predicting 7-year survival than were the ratings of medical professionals.^10^

The Whitehall Study in England demonstrated that changes in health status over time were related to position in the civil service and economy.^11^ This was a large trial with 12,000 patients and produced striking evidence that mortality was directly correlated with amount of environmental stress. That is, heart failure was highest at the lowest levels of the job hierarchy (pressed by everyone) and lowest at the highest levels of the hierarchy (pressed by no one).

A follow-up study used QOL to measure changes in health status over time. The Whitehall-II Study shows that SF-36, when applied to civil services as a study population in 1991 and 1993 and again between 1995 and 1996, demonstrated a difference in health.^12^ The study population was a total of 8349 participants in the earlier study, and 7949 of the participants in the later study completed the entire study.

QOL questionnaires thus became the basis for NSPH. For example, the Behavioral Risk Factor Surveillance Survey (BRFSS) is a telephone survey developed by the Centers for Disease Control and Prevention (CDC), asking 100 health questions to 350K persons per year.^13^ The questions include both general and specific, covering the full range of common lifestyles considered as risk factors, and the persons are demographically representative of the population, using the census to choose samples county by county across the country. The questions are asked by professional interviewers to sample persons chosen by the State Departments of Public Health.

There is an annual survey, but no longitudinal continuity in the persons interviewed. As shown in Figure 1 comparing the BRFSS scale with FHS, demographics is well covered, including age, sex, race, and region, by measuring 100 times the number of persons. The features are similar in scale since FHS included both vital signs (scale of 10) plus medical examinations (scale of 10) versus 100 questions. FHS is the seminal acute care survey, while BRFSS is the seminal chronic care survey. Both infer population baselines from individual variations, using physiological and psychological measures.

## Health Determinants for Full-Spectrum Health Status

**FIG. 2.**
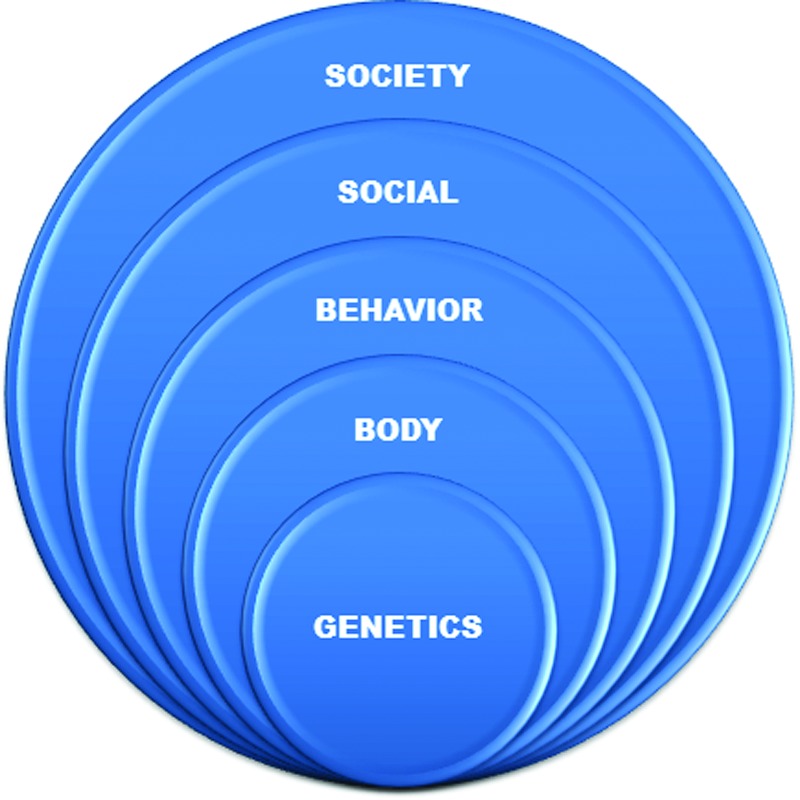
Five rings of status features for human health.

The determinants of health span from the bodies of individuals to the societies of populations. Such determinants can be summarized by a series of concentric rings, as shown in Figure 2, outlining the framework of *Healthcare Infrastructure: Health Systems for Individuals and Populations*.^1^ This book formed the foundation for the 2012 planning workshop for NSF and NIH Smart and Connected Health program on Measuring Data for Population Health,^2^ which discussed challenges in developing large-scale measurement testbeds for population baselines. This workshop, in turn, formed a foundation for the 2015 planning workshop on Mobile Personal Technologies for a Million-Person Cohort of the emerging National Institutes of Health (NIH) Precision Medicine Initiative (PMI). Over the next 5 years, NIH will be funding a Participant Technologies Center to support this prospective PMI cohort. This ring diagram is evolved from the influential study sponsored by the Institute of Medicine, *The Future of the Public's Health in the 21st Century*.^14^ The same viewpoints of health causes within health systems are^15^ behavior 40% (middle), genetics 30% (inner), environment 20% (outer), and healthcare 10%.

**FIG. 3.**
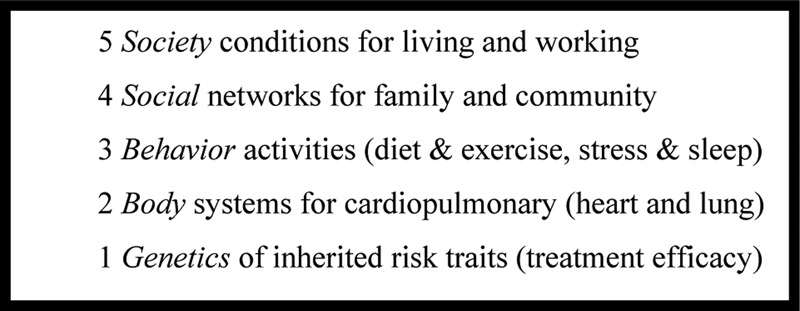
Rings numbered and explained to reflect power and speed.

In measuring health, it is important to include factors ranging from the bodies of individuals to the societies of populations, as shown in Figure 3. These include internal functioning considered by personal medicine, such as genetic inheritance, and vital signs such as heart and lung regulating metabolism and motion. However, it is equally important to include external functioning considered by public health, such as social networks of families and communities, and societal conditions for living and working. These also have strong effects, slower in progress, but greater in impact. In-between internal body and external society is individual behavior of daily life, such as diet and exercise whose effects integrate genes and environment parts into a whole.

Enabling technologies to support this full spectrum exist in research prototypes today. On inner rings, data sensors can simulate pulse oximeters for blood and breath, as well as support gait analyzers for metabolism and mobility. On outer rings, data sensors can locate social interactions, while text parsers can extract personal narratives for societal conditions. In the middle, combinations of data and text in mobile devices can record and analyze the diet and exercise from daily lifestyles. That is, the full range of health determinants must include both physiological and psychological data from daily life.

## Future Technologies for Measuring Health Using Mobile Devices

The problem with health systems is decreasing cost while maintaining quality. The solution with information systems gives data to provide ground truth evidence for making personal medical decisions. Traditionally, such evidence has been electronic medical records (EMRs), but forthcoming technologies can support personal health records covering all the rings. These leverage front end clients using mobile sensors and back end servers using cloud clusters, enabling healthcare infrastructure on the Internet.

Continuous monitoring across all rings will improve healthcare with better predictive modeling. This might be termed the 3M strategy for population health: Monitor–Measure–Manage. To *manage* populations to decrease cost and increase quality, it is necessary to *measure* populations across all the rings to determine health status. To *measure* population health deeply, it is necessary to *monitor* individuals on a continuous basis.^16^

The fitness market has commercialized wearable devices for the inner rings. For example, monitoring armbands can measure ring 2, continuous heart rate and body temperature,^17^ which can then be used to evaluate ring 3 behavior patterns, such as exercise regime and sleep quality. As might be expected from the term fitness, the software focuses on specific health problems such as weight loss for obesity management. Smartphones can already reproduce many of the same measures, such as heart rate using the camera, with millions of downloads for popular phone apps, such as Azumio Instant Heart Rate. The accuracy of phone-based heart rate is limited by the embedded sensors; there are also smartphone attachments, which provide medical quality electrocardiograms for heart rhythm.^18^ High-quality fitness monitors^19^ can measure the R-R rate, the recovery rate between heart beats after exertion, which is a precise measure of fitness.

Digital pedometers measure step counts, which are a simpler measure of daily fitness; these sell in the tens of millions,^20^ but are used by healthy persons rather than chronic patients. Traditional pedometers are small devices clipped to your belt, while newer ones are wristbands, which must compensate for arm movement. Again, smartphones can reproduce such measures with higher accuracy using the embedded accelerometer since the computation for stepping can be personalized to individuals and their situation. Pedometers contain accelerometers, but step thresholds are tuned to typical speeds of healthy individuals, so they undercount steps for chronic patients, who require personalized models.^21^

Mobile devices can also help relate genes to environment. Wearable sensors have measured relationships of internal rings to external rings, such as the heart rate to physical location.^22^ For example, time spent outdoors in green places such as parks correlates with better metabolic rates. Social interactions are characteristic of individuals within populations since they relate behavior to environment. For example, health behaviors can be predicted by simple interactions of social contacts measurable with smartphone sensors, such as personal exposure versus weight gain.^23^

Stress in daily life can be measured using wearable devices containing multiple sensors.^24^ Such psychological stress reflects behaviors more correlated with outer rings than with inner rings and has proven hard to measure accurately with such single sensors as galvanic skin response. Multiple signatures of measures such as heart rate and respiration pattern are accurate in predicting total stress,^25^ showing the utility of combining multiple vital signs for continuous health monitoring.

**FIG. 4.**
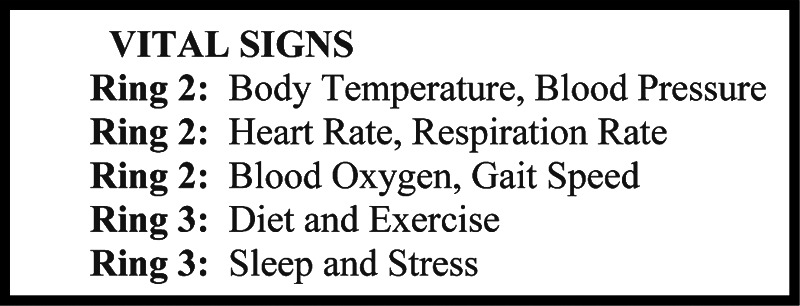
Vital signs recordable by health monitors.

The sweet spot in the near future appears to be smartphones, whose sensors can approximate many measures for vital signs, as summarized in Figure 4. Phone sensors can support health monitors, including inner rings using the camera and microphone for heart rate and respiration pattern, outer rings using the GPS for location stress and social interaction, and middle ring using the camera and accelerometer for diet and exercise. Mobile devices can accurately detect body motion and personal surroundings.

In particular, smartphones contain accelerometer sensors, as do high-end music players. For example, an iPhone and an iPod Touch have the same motion sensors. These sensors can be used to accurately record walking patterns, including computation of gait speed,^26^ which is a special case of activity recognition.^27^ Gait motion analysis is widely applicable to detect abnormal health situations as a continuous window into body function.^28^ In the future, gait analysis will be supported by every mobile device beyond the step counting supported by the current generation of mobile operating systems. This is clinically important since gait speed is closely correlated with senior mortality^29^ as well as chronic status for major conditions, such as heart disease^30^ and lung disease.^31^ For example, oxygen saturation is the single most important clinical measure and is closely correlated with walking motion for cardiopulmonary patients. Surprisingly, this implies that an appropriate predictive model can accurately measure clinical stability using only phone sensors.^32^

Internet Health Monitors (IHM) are new information technology that can be used to measure and to manage individuals and populations.^33^ A situation in healthcare consists of a particular individual with particular demographics who has a particular diagnosis and a particular treatment. The promise of IHM for chronic illness^34^ is that continuous monitors can detect abnormal situations rapidly enough for preventative treatment and effectively enough for care routing to appropriate providers. This can be accomplished in scalable manner by using mobile devices connected to Internet services now averaging more than one device per person worldwide^†^ and already widely used for health monitoring.^35^

## Adoption Strategies for Population Monitoring Using Mobile Phones

The big advantage of mobile phones is that they are already carried for other reasons by the entire population. Thus, they can be effective for population measurement without the need for sociological change. A passive monitor using motion sensors requires no change in behavior for the individual. Thus, the barrier for adoption is low. Phones have a valuable niche already as communication devices, so they are more likely to be frequently used compared with more specialized devices that must be specifically utilized, such as fitness bands. These tend to be used for some period, then discarded, whereas phones seem to have staying power for their other uses.

Note also that at present, smartphones and smart watches have adequate sampling for gait analysis comparable with the quality of expensive medical devices costing an order of magnitude more. However, fitness devices worn on the wrist or waist are standalone packages with long-lasting battery life. Thus, their sampling rate is limited to save power and they cannot currently measure steps or support gait analysis with the clinical accuracy of medical devices.^21^

Mobile phones are communication devices at their core. This means that in addition to their utility as portable sensors, they can be utilized as data collectors for personal devices. Phones as hubs have been the primary transformation thus far of health monitors at the level of individuals, particularly for the requisite maintenance of chronic conditions. For example, implanted monitors for diabetes management can send alerts to smartphones to notify individuals of low blood sugar, indicating the need for insulin dosage, or steroid inhalers for asthma management can correlate doses with locations by using GPS sensors and alert patients when they are entering areas that may trigger their condition. Such devices are of course limited to the individuals who have the condition, plus are willing and able to carry such additional devices to manage their condition.

More useful for population scale are the consolidators for multiple devices, which make it convenient for patients to manage their health through their phones. Most recently, such consolidators have been built directly into the operating systems, for example, the Apple HealthKit stores measures from many external monitor devices onto iPhones for later analysis. Building on this, the Apple ResearchKit also supports obtaining consent for clinical trials. This middleware has enabled a number of trials for monitoring chronic diseases at the scale of thousands of patients, demonstrating the willingness of individuals to participate in population measurement by sharing their personal data.

If more intrusion into patient lives is acceptable, then social analysis can be supported by inferring social interactions from phone usage. That is, some measure of volume and variety of social interactions can be inferred from call logs and location tracking. Social interactions are correlated with prevalent conditions of mental stress, such as anxiety and depression. For example, ginger.IO is the commercial spinoff of social research mentioned above,^23^ now supporting screening for mental stress for hundreds of thousands of patients. Note that privacy becomes a problem since the phone is tracking the person's actual activity. With motion sensors for gait analysis, it is possible to maintain privacy by sampling signals to disguise personal identity.^36^ If more personal information is provided by recording diaries, then deeper context is available, as with the Kavli HUMAN Project highlighted in this Journal.^37^

Most seniors, the oldest population with the greatest need for healthcare, today do have mobile phones, but generally feature phones rather than smartphones.^35^ Smartphones are still distinguished by touch screens, so they have motion sensors to detect screen orientation. Feature phones do not have these sensors, so they cannot be utilized for the all-important gait analysis. They can support diet monitoring, rather than exercise monitoring, by using the camera to record meals for nutritional analysis as well as the stress monitoring just mentioned. Over time, the features in smartphones have migrated into feature phones. In the next generation or two, most sensors needed for health monitoring will be available in feature phones, for example, in less than five years, the cheapest mobile phones will have motion sensors. This is important since predictive models for gait analysis are equally accurate with the cheapest smartphones^21^ due to walking being so slow compared with processing. In developing countries, phone logs have already been utilized to track epidemics.^38^ In the near future, the healthcare infrastructure of developing countries will be based upon NSPH with cheap smartphones everywhere for scalable monitoring.

## National Scale Using Internet Services

The largest website in the first decade of the Web in the 1990s was Yahoo, which provides a general portal to a wide variety of information on the Web. For over a decade, this portal has served more than 350M unique users each month with personalized content optimization, where the stories and the layout of its services and its news are different for each group.^39^ The groups are daily updated clusters of similar persons, which essentially reflect population cohorts based on features and demographics. Analysis of usage data sets enables information analysts to interactively select optimal groups after statistical clustering on international hierarchies of cloud computers. This large-scale commercial service is a realistic model for the new healthcare infrastructure with population cohorts determined interactively after supercomputer analysis. A healthcare version would need to gather different data about health status of bodies instead of click status of topics, but the basic architecture would be similar.

Yahoo has been passed in scale by two other websites, both supporting more than 350M different users each day from more than 1B total users.^40,§^ The timescale for the later decades of the Web is compressed so that in 2014, Yahoo is 20 years old, Google is 15 years old, and Facebook is 10 years old, and there are now a number of newer companies projecting to operate at this same scale when including international users.

Google became prominent as a search engine, which gathers Web documents from other sites and rank orders the results corresponding to user-specified query words. However, their business model is actually targeted advertisements, using their knowledge of the user and the query to display advertisements. Google's user model is today's deepest knowledge of living and working conditions, which is gathered by collecting personal messages from e-mail and voice mail among other sources. These represent Ring 5 data at the requisite scale when the personal narratives from the recorded messages are extracted.

One example where search logs have already been effective in healthcare is the use of Google Trends to track epidemics, similar to the call logs mentioned above. Since location can be inferred from network addresses, search terms related to flu can be correlated with specific regions and track incidence for treatment strategy. The automatic volume of Flu Trends could make up for the inaccuracy compared with the manual method of physician reporting of flu cases. Search logs are not adequate for health monitors since they reflect global interest, not local activity, as shown by confounding effects of people in regions with little flu reading articles about people in regions with much flu.^41^ Carried phones can accurately monitor personal status, but the same people can read about other conditions of other people over the Internet.

Facebook became prominent as a social medium, which enables persons to post images and text to share with user-selected friends and communities. Their internal statistics record the social interactions within relationship networks of families and friends, from which both quantity (frequency) and quality (tie-strength) can be derived. These represent Ring 4 data at the requisite scale. Note again that the scale of persons is as required for healthcare infrastructure, but the current scale of features is lower.

Facebook demonstrates that billions of persons are willing and able to post frequent updates to personal status, provided they have streamlined software. Adoption of similar strategy into healthcare infrastructure could enable widespread availability of personal diaries that log health status and relevant situations. Facebook usage is now dominated by mobile devices; there is widespread usage even in developing countries where mobile phones with data plans are often sold for Facebook, not for Internet. Other popular social media, such as Twitter, directly support the posting of status to interested groups. Again there is an analogy into healthcare infrastructure for treatment outcomes into population cohorts. Tweets are too short for effective extraction of health status, but health messages from online forums are often several paragraphs, providing enough context. For example, Yahoo Groups mentioned above has been utilized to automatically extract adverse effects of common drugs for common diseases,^42^ using such text mining and natural language processing techniques as sentiment analysis.

As noted above, demographic variation requires about 100 times the Framingham number or about the scale of BRFSS of 350K. The primary features for demographics are age, sex, race, and region. There are roughly five age groups and 2 sexes, where ages are teenager 10–19, college 20–29, adult 30–49, boomer 50–64, and senior ≥65 years. There are roughly five races as counted by the US Census and seven regions as US populations within the Bell System regional operating companies. The computation for 350K is 5 × 2 × 5 × 7 = 350 cohorts of 1000 persons each, with replicates for individual variation.

To handle all diseases and conditions, another factor of 1000 is needed to survey broad status about health risks and then to survey deep connections with all disease conditions. One thousand is the scale of the main categorization for modern disease classifications,^43^ such as International Classification of Diseases, ninth revision (ICD-9), or the Merck Manual.^44^ Historically, 1000 has been the scale of each system of medical diagnosis from India and China to Greece and Rome (see chapter 2 of Ref.^1^). The 1000 categories are likely a fundamental feature of human memory, roughly three hierarchical levels with 10-way branching, as shown by library classifications such as subject thesauri such as MeSH for medicine or INSPEC for engineering. Thus, to support all situations with healthcare infrastructure, the health monitors need to frequently track 350M persons.

The requisite scale of population baseline is happily the same scale as national surveys. The 2015 estimated US population is slightly over 320M, growing at 0.73% per year.^45^ Thus, within this decade, a national-scale health survey in the United States could cover full-spectrum health status for population baseline analysis. Actually, since the 1000 conditions are overlapping to some extent, the scale is already sufficient. A decade ago, handling national-scale daily interactions would have been infeasible. Today, it is already routine for the most popular websites with major server farms. A decade hence, it will be routine for specialized servers over the Internet then. The distributed technology is already commercial.

The scale of features at present is far less than the requisite scale, however. Google might know 100 things about you, inferred indirectly from multiple searches. Facebook similarly might know 100 features, entered directly as part of your profile for sharing. This is the same scale as the BRFSS, the 100-question current national population survey, although the topics are different. To transform the current 350K-person demographics measured annually into the future 350M-person lifestyles measured daily, the health features across all status rings must also be gathered on a daily basis. For example, 10 vitals × 10 values × 300 days are 30K features. The vital signs as listed in Figure 4 are the major health features that change quickly enough to be worth measuring their daily status. These focus on Ring 2 and Ring 3 with a bit of Ring 4 since Ring 1 and Ring 5 change more slowly than each day.

## National Scale Population Health Surveys

Today it is technically feasible to support national-scale population surveys. As noted, multiple websites have more than 350M users, although they do not cover every person in the United States. What have become basic services of search (Google) and sharing (Facebook) are predominant in this regard. Conveniently, national scale also encompasses the population variation that is necessary to predict health status in everyday life. This would move beyond the demographic scale to disease scale to cover enough features for enough persons to predict health status on a daily basis. Then, any person who is continuously monitored can be assigned a demographic group with disease cohorts and risk assessment computed by correlation with these cohorts. Rather than manually diagnosing an individual patient with a condition for treatment, the feature vector for the patient could be automatically utilized to cluster into a population cohort. Then, the treatments most effective for that cohort could be prescribed, via these automatic guidelines.

A National Survey of Population Health would measure health status of every person every day. Given the penetration of mobile phone usage at 90% in the United States, a US survey similar to the BRFSS would be technically feasible, with suitable inducement. The penetration of Internet services shows similar interaction at similar scale. Of course, mobile phones have the advantage that the measured persons are already connected to a national network and that no additional devices are necessary for the measures.

Assuming that daily status can be accurately monitored at national scale, what predictions can be made from status models to effectively and efficiently support diagnosis and treatment? A straightforward application would be for the model to generate a 1000-vector containing the risk/status of each disease/condition. Then, for a desired demographic, a match into disease cohorts is made to obtain risk for each. For example, a common claim for the utility of personalized medicine is risk assessment from genomic screening, a disease analog of the risk factors from demographic analysis.

**FIG. 5.**
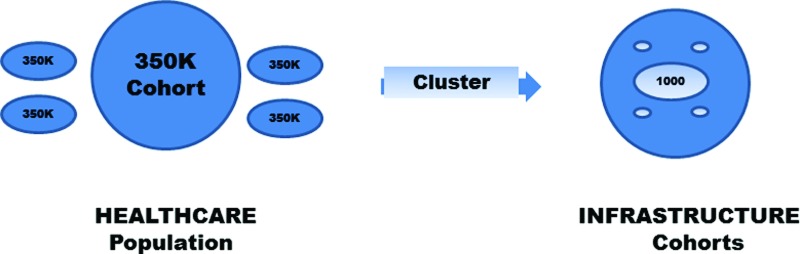
Clustering similar patients in feature space produces monitored cohorts. At the healthcare level, the national pool of 350M individuals is partitioned into population cohorts of 350K persons each, where each cohort is clustered in 30K-dimensional feature space of health determinants. The clustering is thus feature derived rather than disease derived. At the infrastructure level, each cohort is now reduced to the scale of present national surveys, where there are 350 demographic cohorts of 1000 persons each. That is, each light-colored shape represents the human variation of 1000 persons with similar health features and similar population demographics, so they are sufficiently accurate to discover effective treatments.

A more population-centric prediction would be modeled upon cluster analysis from information retrieval. This is compute intensive, requiring large parallel computers to discover similarity clusters in n-space by matching 1000 different groups of 350K persons from the pool of 350M. By clustering in 30K-dimensional feature space using health determinants, the closest groups to the specific person can be computed. These 1000 cohorts are not disease derived, but feature derived, representing groups of persons whose health status is closest to particular individuals. This computation effectively reduces the future national surveys at 350M scale to multiple versions of the present national surveys at 350K scale, based upon digital health population measurement. As shown in Figure 5, predictions for effective treatments for current status can be provided by summarizing the treatments of the closest cohorts in feature space to the health vector for the particular individual. This would provide a dynamically generated support group, analogous to those manually selected groups proven effective for treatments of chronic disease.^46^

Effective treatments for future status can be provided by using the health monitors to detect deviation against cohort baseline as a continuous metric for determining when diagnosis and treatment might need to change. Supporting this requires establishing baselines for population cohorts by measuring appropriate size samples for each monitored cohort with clinical validations.^47^ Deviation detection of abnormal status can be supplemented by point-of-contact diagnosis. When a monitor detects deviation from normal baseline, persons can be queried to record detailed self-reports of their condition. The logistics are eased by voice interactions using the carried phones. These self-reports are a point-of-contact version of health messages posted into online forums by patients seeking advice. Text mining of health messages has shown it feasible to cluster treatment effectiveness into which persons have which outcomes.^48^

NSPH require cohort analysis after the health monitors have generated continuous records of health status. The BRFSS is implemented state by state through the statewide Departments of Public Health. These departments contract phone interviewers to administer the questionnaires to a representative sample for each county within the state, with the sample choices determined by the demographics of the census. The questionnaires are developed and the funding is provided centrally through the CDC, who also performs the meta-analysis to identify health trends.

Distributed systems with central control are almost certainly necessary to support NSPH, through the use of hierarchical networks where each subregion can operate independently, then be combined into larger regions, to eventually support national scale. Since health monitors focus on patient status, it might be preferable to be implemented through regional health systems since they link into medical records and treatment providers. A typical system will cover 350K persons across its region. It is well known that there is significant regional variation in healthcare diagnosis and treatment.**

The national implementation of EMRs has followed this regional strategy, where the Regional Health Information Exchanges (RHIEs) federate records from each health system within a region,^49^ then the RHIEs are combined at the national level with central coordination from the Federal Office of the National Coordinator (ONC). Since there are nearly 1000 health systems in the United States,^50^ with widely varying EMRs, the federation is necessarily inaccurate, requiring federating together the most equivalent fields across databases, even though the semantics differ.

The Internet itself is a federated hierarchical network, but at a much lower level of network protocols, namely packet transmission with Internet Protocol. The search capability of Internet services has already transformed from syntactic to semantic interactions as information retrieval has deepened.^51^ Internet services providing semantic interactions, such as search or sharing, have similarly been most successful by implementation of distributed systems via a single controlling entity.^52^

A national-scale health survey would be the 21st century version of the BRFSS, thus a suitable responsibility for the CDC, as funded by the Federal Government in the United States. Alternatively, another federal agency could be created, as proposed by several introduced National Health Tracking bills in the US Congress, which would assess the general health status of the US population by monitoring more deeply than Healthy People 2020. This agency might be modeled after the Patient-Centered Outcomes Research Institute (PCORI), which gets a percentage of Medicare monies for R&D purposes, but is an independent agency. Health insurance is a natural pathway toward national-scale surveys since inducements can be given both by carrot, such as discounts, and by stick, such as requires. Public agencies such as Medicare can require periodic compliance with health monitors to provide care, whereas private entities such as Kaiser can provide lower costs for persons who monitor frequently.

National Surveys of Population Health will revolutionize the practice of evidence-based medicine^53^ in the 2010s as the concrete realization of information technologies envisioned only in outline^54^ during the 1990s. For generic measurement at population scale, paper questions will finally be replaced by mobile sensors. Viable healthcare is possible with modern technology, offering a way out, a way beyond the economic crisis of our time.

## Abbreviations Used

BRFSS: Behavioral Risk Factor Surveillance Survey
CDC: Centers for Disease Control and Prevention
EMRs: electronic medical records
FHS: Framingham Heart Study
IHM: Internet Health Monitors
NIH: National Institutes of Health
NSPH: National Surveys of Population Health
PMI: Precision Medicine Initiative
QOL: Quality of Life questionnaires
RHIEs: Regional Health Information Exchanges
SF-36: Short Form 36

## Notes

^†^ According to Gartner, 1857M mobile phones were shipped in 2013, dominated by Nokia. According to Juniper Research, 980M smartphones were shipped in 2013, dominated by Samsung. UNESCO estimates that there are 6B phones with mobile subscriptions worldwide in 2013, averaging more than one per person worldwide.

^§^ Facebook reported reaching 1B registered users on September 14, 2012. It reached this milestone in 8 years, while Google took 13 years to reach 1B users, as achieved in 2011. According to Experian Hitwise, in February 2014, Google has 2.8B and Facebook has 2.3B weekly visits or about 350M distinct visitors daily.

^**^ The Dartmouth Atlas has documented for over 20 years the regional variations in cost and quality of American healthcare (www.dartmouthatlas.org/).
